# Feasibility of the Remote Physical Activity Follow-Up Intervention after the Face-to-Face Program for Healthy Middle-Aged Adults: A Randomized Trial Using ICT and Mobile Technology

**DOI:** 10.3390/ijerph19084922

**Published:** 2022-04-18

**Authors:** Koji Yamatsu, Kenji Narazaki

**Affiliations:** 1 Faculty of Education, Saga University, Saga 840-8502, Japan; 2 Graduate School of Advanced Health Sciences, Saga University, Saga 849-8501, Japan; 3 Graduate School of Medical Science, Saga University, Saga 849-8501, Japan; 4 Center for Liberal Arts, Fukuoka Institute of Technology, Fukuoka 811-0295, Japan; narazaki@fit.ac.jp

**Keywords:** behavior change, non face-to-face, ehealth, mhealth

## Abstract

Although the effectiveness of face-to-face and remote intervention for increasing and maintaining physical activity (PA) have been compared, the effect of combining the two forms of intervention is unknown. The purpose of this study was to examine the feasibility of the remote PA follow-up intervention after the face-to-face PA program on changing PA behaviors and some health outcomes in healthy middle-aged adults. As a secondary analysis, we also attempted a preliminary analysis of the difference in the number of behavior change interviews in the remote PA follow-up intervention. After the face-to-face intervention, 30 healthy subjects were randomly divided into four behavior change coaching interviews (BCI4 group) or three BCI (BCI3 group). The results of this study showed that body weight, body fat mass, and waist circumference were significantly reduced after face-to-face intervention, and were further reduced after remote PA follow-up intervention. However, the difference in the number of BCI affected only body fat mass. The remote PA follow-up intervention may have potential to maintain the effects of face-to-face intervention. In the future, it is necessary to refine the research design and conduct a full-scale intervention study.

## 1. Introduction

Regular physical activity (PA) is a high-priority lifestyle-related measure for the prevention and improvement of non-communicable diseases such as cancer, heart disease, and diabetes [1].

The implementation of PA interventions typically involves either face-to-face or remote (non-face-to-face) methods, the relative effects and efficacy of which have been studied [2]. Face-to-face intervention often takes the form of participants meeting with an instructor. Previous studies reported that participants in face-to-face interventions were highly motivated to change their behavior during and immediately after the intervention period, but often became less motivated and unable to maintain their behavior change [3]. By contrast, remote intervention is a form that is provided without directly meeting the instructor and via telephone or the internet. Although remote intervention has the advantage of convenience for the subject, its behavior-changing effects have been reported to last only briefly [4].

Remote intervention has been dramatically changed in recent decades, owing to remarkable progress in communications and personalized advice technologies. Initially, mail [5] and telephoning [6] were the communication methods used for remote interventions, but these have been replaced with the widespread adoption of new information and communication technology (ICT) methods such as the internet and smartphones. Remote intervention using ICT has several advantages compared to face-to-face or previous non-ICT methods: for example, fewer location- and time-based constraints than face-to-face meetings, lower costs than conventional mail and telephone, and computer-tailored or personalized support [7].

While previous studies have compared the effectiveness of face-to-face and remote intervention methods [2], further research is needed to illuminate how they should be combined in the future. In addition, the findings of intervention studies when remote PA interventions are continued after face-to-face interventions are extremely limited.

In this study, we sought to clarify how face-to-face and remote interventions can be combined to be more effective in changing behavior. This is the first study to investigate the hypothesis that remote interventions can maintain and improve the effects of face-to-face interventions. Although numerous studies have compared face-to-face and remote interventions in parallel, none to date have assessed their combination in this manner. Consequently, this research has valuable potential for both public health and the healthcare industry.

### Purpose

The purpose of this study was to examine the feasibility of remote PA follow-up intervention after a face-to-face PA program on changing PA behaviors and some health outcomes in healthy middle-aged adults. As a secondary analysis, we also attempted a preliminary analysis of the difference in the number of behavior change interviews in the remote PA follow-up intervention.

## 2. Materials and Methods

### 2.1. Study Design

**Figure 1:**
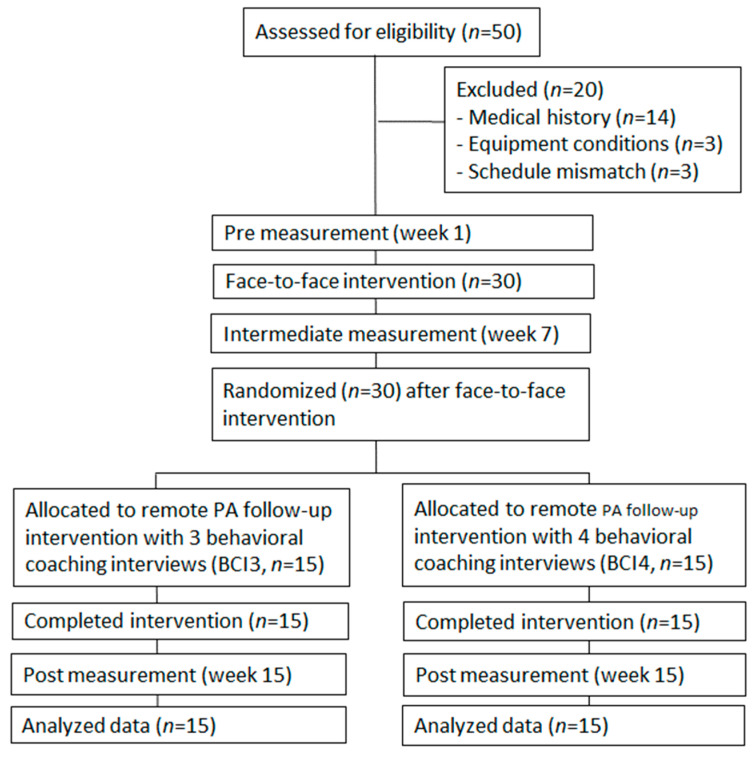
Study flow.

This study adopted a randomized trial design, as shown in Figure 1. All subjects received a face-to-face PA intervention, followed by remote interventions. After the face-to-face intervention, the subjects were randomly divided into two groups according to the number of behavior change coaching interviews (BCI) in the remote PA follow-up intervention. The first group received four BCI (BCI4 group), and the second received three BCI (BCI3 group). Randomization allocation was stratified to make the number of participants equal. The remote PA follow-up intervention took the form of a behavior modification program aimed at changing PA levels via the internet. Both groups received almost equivalent interventions; but the only difference was the number of BCI.

### 2.2. Recruitment

The participants were recruited through various forms of public advertising (homepage, city bulletin, posters, etc.) in Yukuhashi city, Fukuoka Prefecture in Japan. The conditions for participating in this study were that participants should: (a) live or work in this area; (b) be in their 50s or 60s; (c) have a device, such as a smartphone that can access the internet; and (d) consent to participate in the program. The recruitment poster stated that specialists would provide exercise support both face-to-face and remotely.

### 2.3. Eligibility Screening and Consent

Of the 50 candidates, 30 subjects were selected as participants. Of the 20 subjects who were excluded, 14 were disqualified due to illness or medical history, 3 did not possess the required equipment, and 3 had scheduling constraints that made them unable to participate fully in the study.

To ensure the safety of the study subjects, those who had been diagnosed with the following conditions by a doctor were not allowed to participate in the study:

- Heart disease: heart failure, aortic stenosis, dilated cardiomyopathy, aortic aneurysms, aortic dissection, Marfan’s syndrome, myocarditis, long QT syndrome, unexpected metroendometritis, pacemaker, IDC (implantable cardioverter defibrillator), severe arrhythmia/unstable angina;
- Pulmonary disease: chronic respiratory deficiency;
- Diabetes: fasting blood glucose level 200 mg/dL or more, HbA1c 10% or more, occasional blood glucose level 300 mg/dL or more;
- Renal failure, artificial dialysis, cirrhosis, acute hepatitis, or pancreatitis.

Candidates with the following diseases were allowed to participate in the study only with their doctor’s permission:

- Heart disease: myocardial infarction, cardiac hypertrophy, stable angina, heart stent heed. heart valve disease, congenital heart disease atria, ventricular septal defect, arrhythmia;
- Brain disease: cerebral infarction, cerebral hemorrhage, subarachnoid hemorrhage, epidural hematoma, etc.
- Cancer, hypertension, diabetes, renal disease, collagen disease, osteoporosis, chronic hepatitis, epilepsy, pneumothorax, gout, deep vein thrombosis, continued taking any medicine, and designated intractable disease.

In addition to the subject’s health status, participation was allowed only if the smartphone or communication device they possessed met the following conditions:

- Apple device (iPad or iPhone), running iOS 9 or later;
- Android (smartphone or tablet) with a camera, running Android 5.0 or later (API level 21 or later);
- Bluetooth compatible.

These conditions were necessary for the remote behavior change support system in this study to operate normally.

### 2.4. Data Collection and Measurement

Three measurements were made to test the hypothesis. The first two measurements were made before (week 1) and after (week 7) the face-to-face interventions. The final measurement was made after the remote follow-up interventions (week 15). The three measurements included assessments of body weight and composition, physical fitness, comprehensive life-related conditions, and psychological distress.

PA and sitting time (ST) were evaluated during the face-to-face intervention (weeks 1 to 7) and during the remote follow-up intervention (weeks 8 to 15) every day.

### 2.5. Interventions

All interventions lasted from 26 October 2019 to 8 February 2020. The face-to-face intervention duration was 26 October–14 December 2019. The remote PA follow-up intervention duration was 15 December 2019–8 February 2020.

#### 2.5.1. Face-to-Face PA Intervention

The face-to-face intervention was provided by RIZAP Co., Ltd. (Tokyo, Japan), which is a famous fitness company in Japan. Face-to-face interventions with instructors were conducted at the city’s athletic facility once per week. All the subjects were divided in half, and the interventions were conducted in the morning and afternoon. The instructor in the face-to-face intervention was qualified for public health exercise guidance in Japan and was in charge of everything alone. The duration of each session was 90 min (60 min for lectures, 30 min for fitness exercises). The sessions in the first and last was 150 min to accommodate time for research measurements (60 min for measurement, 60 min for lectures, 30 min for fitness exercises).

The contents of the face-to-face PA sessions were as follows. In lectures, participants learned about the importance of diet, the method of fat intake, and how to eat a low-carbohydrate, high-protein diet. In the exercise sessions, participants performed stretching to improve flexibility, aerobics to improve endurance, functional training to increase range of motion of joints, and strength training to improve muscle strength, mainly through moderate-to-vigorous intensity PA (MVPA) and bodyweight exercises. The exercise intensity (number and time) in the face-to-face intervention was gradually increased so as not to become excessive. We made every effort to ensure the safety of exercise such as checking health conditions such as blood pressure before starting each exercise.

#### 2.5.2. Remote PA Follow-Up Intervention

The remote intervention was provided via the internet using a remote behavior change support system co-developed by the first author and Seiko IT Solution Co., Ltd. (Fukuoka, Japan). In the BCI4 group, a total of four BCI, lasting about 30 min each, took place within 8 weeks, in week 8 (one week after the start of the remote intervention), week 9, week 11, and week 14. Each BCI was conducted by an expert with a PhD in behavior change. The BCI3 group’s interviews were nearly identical, but occurred only three times, in week 8, week 11, and week 14. The remote intervention took place during the festive season in Japan. Therefore, the number and timing of the BCI were designed such that events such as Christmas and New Year would not prevent continued behavior modification [8].

Each BCI was conducted via a video conference system, with the interviewer and participant able to see each other’s facial expressions. The interviewer could visualize and analyze the Fitbit data automatically collected from each participant (see Section 2.6.3). The first goal of the interview was to identify and discuss the participant’s problems about PA behavior and to determine a behavior change goal [9]. The second goal of the interview was to discuss the participant’s achievement of behavior change goals, and how to cope when their achievement fell short of their targets [9]. The final interview addressed what was needed for the participant to continue changing their behavior after the remote intervention had concluded [9]. The final goal of the BCI was to maintain increased PA in face-to-face programs and, if necessary, to discuss activities that would lead to reduced ST.

**Figure 2:**
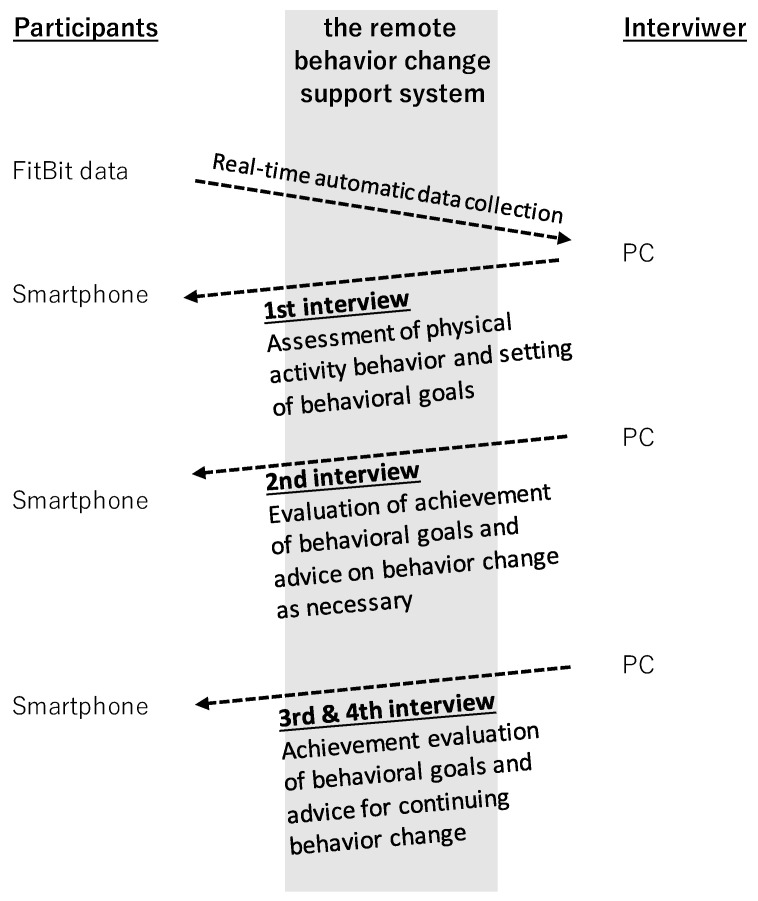
The content/structure of the behavior change interviews.

The remote behavior change support system was the primary tool through which non-face-to-face intervention was provided (see the Figure 2). This system can be divided into parts related to the provision of the BCI, and those related to automatic data collection and display. The main functions of this system were to manage the 30 participants’ interview appointments and to implement a video conferencing system. As for automatic data collection and display, Fitbit data were automatically synced with the participants’ smartphone and then uploaded and stored in the specified database via the internet; the data were then displayed on the interviewer’s screen as a graph. The system contributed to the visualization of PA and ST patterns.

The security of the remote behavior change support system is also important, given the privacy concerns associated with storing and handling the personal information obtained in this research. To protect participants’ privacy, access to the system required an ID and password. Moreover, login information, personal data, and input information were encrypted and sent and received using the Secure Sockets Layer (SSL) protocol. The entire system was protected from unauthorized access by a firewall, and the server was located in the Seiko IT Solution Co., Ltd. data center (ISMS certification: ISO/IEC27001), which operates with strict security controls.

### 2.6. Objective Measures

#### 2.6.1. Weight and Body Composition

Body weight, body fat mass, and skeletal muscle mass were measured with a Tanita DC320 (Tanita Co., Ltd., Tokyo, Japan). Body fat mass and skeletal muscle mass were estimated by the impedance method in a Tanita DC320. In order to adjust for the weight of participants’ clothes in winter, 1 kg was subtracted from the measured value. The waist circumference was measured horizontally above the participant’s navel with both hands extending to the left and right of the body, and while exhaling.

#### 2.6.2. Physical Fitness

Grip strength was measured by a mechanical dynamometer on the left and right, and the maximum value of trials was used for analysis [10]. Maximum walking speed was obtained by asking the participants to walk a distance of 11 m at the speed of their maximum effort and measuring the time required for 5 m on the way. The value in m/s was calculated by dividing 5 m by the time in seconds taken to walk 5 m [10].

#### 2.6.3. Physical Activity and Sitting Time

PA and ST were measured by a wearable device (Fitbit Inspire HR, Fitbit). The Fitbit Inspire HR has been confirmed to be valid and reliable, along with the earlier Fitbit Alta HR [11]. All participants were lent a Fitbit device for free and were instructed to wear it on their wrist at all times during all interventions. They launched a dedicated app on the smartphone once every few days and were asked to update the data. The Fitbit measured MVPA (min/day), low intensity PA (LPA, min/day), walking steps (steps/day), and ST (min/day). For all indicators, only the data for days when the devices were worn for 10 h or more were used, following previous studies [12,13,14].

### 2.7. Subjective Measures

#### 2.7.1. Comprehensive Life-Related Conditions

The Kihon Checklist (KCL) was developed by the Ministry of Health, Labor and Welfare in Japan as a tool for the early detection of elderly people who are likely to need long-term care [15]. The KCL consists of 25 items (yes/no) divided into seven domains: daily life-related movements, locomotorium, malnutrition, oral function, withdrawal, cognitive function, and depressed mood. The KCL score is a continuous variable with a maximum of 25 points. The higher the total KCL score, the higher the risk of long-term care.

#### 2.7.2. Depression and Anxiety Symptoms

Kessler’s six-item psychological distress scale (K6) was used [16,17]. The validity of the Japanese version of K6 has been verified [18]. The K6 score ranges from 0 to 24 points, calculated determined by participants’ answers to six items rated on a 5-point Likert scale; the higher the K6 score, the more severe the subject’s psychological distress [16,17].

### 2.8. Statistical Analysis

For the analysis of body composition (body weight, body fat mass, skeletal muscle mass, waist circumference) and physical fitness (grip strength, maximum walking speed), a two-factor analysis of variance (ANOVA) was used based on the intervention group (BCI4 group vs. BCI3 group) and the measurement time (week 1, week 7, and week 15).

For the analysis of PA and ST, a two-factor ANOVA was performed using the intervention groups (BCI 4 group vs. BCI 3 group) and the intervention duration (during the face-to-face intervention (weeks 1 to 7) vs. during the remote follow-up intervention (weeks 8 to 15)).

All analyses were performed using the Japanese version of SPSS Statistics 27 (IBM, Armonk, NY, USA). When the main effect was statistically significant in the two-factor ANOVA, a multiple comparison test using the Bonferroni method was performed. When a significant interaction was observed, a simple main effect subtest was performed. The significance level was less than 5%.

### 2.9. Human Subjects

The study was approved by the Institutional Review Board of the Faculty of Education in Saga University (research no. SGED0003). All participants gave written informed consent. The trial was registered in the UMIN-CTR in Japan with the protocol number UMIN000041635.

## 3. Results

### 3.1. Baseline Characteristics

Of the 30 subjects, 12 were male and 18 were female. The average age was 59.4 years old, with four males in their 50s and eight in their 60s, ten females in their 50s, and eight in their 60s. There were five males and ten females in the BCI4 group, and seven males and eight females in the BCI3 group, and there was no significant difference in the average age and gender ratio between the two groups. None of them dropped out of face-to-face and remote PA follow-up interventions.

As Table 1 shows, no significant differences in body weight, body fat mass, muscle mass, waist circumference, grip strength, maximum walking speed, KCL score, and K6 score during the face-to-face intervention (weeks 1 to 7) were observed between the BCI4 and BCI3 groups.

There were no significant differences between the BCI4 and BCI3 groups in MVPA, LPA, walking steps, and ST at week 1, week 7, and weeks 1 to 7.

From the above, we can infer that the random allocation divided the two groups equally.

### 3.2. Changes in Body Weight and Composition

The results of analysis of body weight, body fat mass, muscle mass, and waist circumference are shown in Table 2.

A two-factor ANOVA for body weight revealed a main effect of time (F = 37.5, *p* < 0.001), but the main effect of both groups (F = 0.01, *p* = 0.917) and the interaction (F = 1.57, *p* = 0.216) was not significant. As a result of conducting a multiple comparison test due to the significant main effect of time, body weight significantly decreased from week 1 (64.8 ± 12.2 kg) to week 7 (62.0 ± 11.1 kg), that is, during the face-to-face interventions, and decreased further to week 15 (60.7 ± 11.1 kg) after the remote PA follow-up interventions.

The analysis of body fat mass had a main effect of time (F = 22.1, *p* < 0.001) and an interaction effect (F = 3.41, *p* = 0.040), but the main effect between both groups (F = 0.66, *p* = 0.422) was not significant. Since an interaction was observed, a simple main effect test was conducted. It revealed that body fat mass in the BCI3 group was significantly reduced from week 1 (19.4 ± 5.8 kg) to week 7 (17.3 ± 5.6 kg) and was decreased further to week 15 (16.2 ± 5.6 kg). However, there was no significant difference in the BCI4 group.

The waist circumference analysis showed a main effect of time (F = 48.0, *p* < 0.001), but the main effect between both groups (F = 0.01, *p* = 0.918) and the interaction effect (F = 2.85, *p* = 0.066) were not significant. When a multiple comparisons test was performed on the main effect of time, overall waist circumference decreased significantly from week 1 (90.6 ± 10.2 cm) to week 7 (87.2 ± 9.5 cm) and decreased further to week 15 (84.9 ± 9.2 cm).

Muscle mass had a main effect of time (F = 36.8, *p* < 0.001), but the main effect between both groups (F = 0.22, *p* = 0.640) and the interaction (F = 0.41, *p* = 0.664) was not significant. When a multiple comparisons test was performed on the main effect of time, overall muscle mass decreased significantly from week 1 (42.4 ± 9.2 kg) to week 7 (41.3 ± 8.6 kg) and decreased further to week 15 (40.7 ± 8.5 kg).

### 3.3. Changes in Physical Fitness, Life-Related Conditions, and Depression

Table 2 shows the changes observed in participants’ physical fitness, life-related conditions (KCL score), and depression (K6 score). The analysis for grip strength revealed a main effect of time (F = 6.72, *p* = 0.002), but the main effect between both groups (F = 0.06, *p* = 0.807) and the interaction (F = 1.37, *p* = 0.264) were not significant. After performing a multiple comparison test, overall grip strength did not significantly increase from week 1 (32.9 ± 10.5 kg) to week 7 (33.6 ± 10.6 kg), but there was a significant increase from week 1 to week 15 (35.0 ± 10.2 kg).

The analysis of maximum walking speed showed a main effect of time (F = 50.2, *p* < 0.001), but the main effect between both groups (F = 2.94, *p* = 0.098) and the interaction (F = 0.62, *p* = 0.540) were not significant. After performing a multiple comparison test, overall maximum walking speed was significantly increased from week 1 (2.33 ± 0.37 m/s) to week 7 (2.83 ± 0.35 m/s), and this increase was maintained at week 15 (2.90 ± 0.49 m/s).

The KCL score analysis revealed a main effect of time (F = 5.94, *p* = 0.005), but the main effect between both groups (F = 1.50, *p* = 0.231) and the interaction effect (F = 1.22, *p* = 0.303) were not significant. After performing a multiple comparison test, the KCL scores overall were not significantly reduced from week 1 (3.0 ± 2.4 point) to week 7 (2.0 ± 1.6 point), but week 15 (1.8 ± 1.9 point) showed a significant reduction from week 1.

In terms of K6 score, there was a main effect of time (F = 7.19, *p* = 0.002), but the main effect between both groups (F = 0.07, *p* = 0.792) and the interaction (F = 0.46, *p* = 0.631) was not significant. A multiple comparison test showed that K6 scores were not significantly reduced from week 1 (1.8 ± 2.2 point) to week 7 (1.1 ± 1.4 point), but the scores at week 15 (0.6 ± 1.2 point) were significantly reduced from week 1.

### 3.4. Changes in Physical Activity and Sitting Time

As Table 3 shows, there were no main effects of time or between both groups on MVPA, LPA, ST, and walking steps. MVPA (56.3 ± 38.3 to 52.8 ± 38.4 min/day), LPA (292.4 ± 80.4 to 290.1 ± 94.3 min/day), ST (700.3 ± 144.4 to 705.2 ± 151.8 min/day) and walking steps (12,274 ± 4063 to 11,971 ± 4306 steps/day) were not significantly changed. Thus, we can infer that PA and ST were maintained from week 1 to 7 (during the face-to-face intervention) to week 8 to 15 (during the remote intervention). Moreover, no interaction was observed in any analysis.

## 4. Discussion

### 4.1. Summary of Principle Results

The main question in this study was: “Is remote PA follow-up intervention feasible after face-to-face program?” The results of this study showed that body weight, body fat mass, and waist circumference were significantly reduced after face-to-face intervention, and were further reduced after remote PA follow-up intervention. In other words, the weight-loss effects of face-to-face intervention were not only maintained by the subsequent remote PA follow-up intervention, they may be further strengthened. In addition, physical fitness, life-related conditions, and depression were confirmed to be significantly improved after the remote PA follow-up intervention (week 15), but not after the face-to-face intervention. Furthermore, PA and ST during the face-to-face intervention did not decrease significantly during the remote PA follow-up intervention. Thus, the PA level during the face-to-face intervention was maintained even during the remote PA follow-up intervention. These results suggest that a short period of remote PA follow-up intervention after the face-to-face program is feasible and may contribute to PA maintenance, weight loss (prevention of lifestyle-related diseases), and improvement of KCL score (prevention of long-term care).

Significant weight loss was observed in this study, but there was also an unintended side effect of loss of muscle mass. It is considered unavoidable to reduce not only fat mass but also skeletal muscle mass during weight loss. In an attempt to counter this possibility, in this study, we actively recommended a low-carbohydrate, high-protein diet, thus to prevent the participants’ dietary intake being reduced too much during both the face-to-face and remote follow-up intervention. Since it would be ideal to reduce body fat without losing muscle mass, the decrease in muscle mass was 2.6% at week 7 and 4.0% at week 15 compared to before the intervention, which was smaller than the body fat mass decrease of 7.5% at week 7 and 11.6% at week 15. That is, it may be considered that body fat was relatively more reduced. Further research is necessary to find a system by which remote and face-to-face interventions might be combined to achieve this result.

The second question in this study was: “Does the difference in the number of BCI (behavior change coaching interviews) influence the feasibility of remote PA intervention?” The results showed that the difference in the number of BCI affected only body fat mass. The decrease in body fat mass in the BCI3 group (3.2 ± 2.9 kg reduction) was significantly larger than that in the BCI4 group (1.4 ± 2.1 kg reduction). The reason for this result remains unclear. Although the difference in the number of interviews in this study was realistic and clinically unavoidable, the no intervention group that did not provide interviews should be set as described later due to the limitations of the study.

### 4.2. Comparison with Existing Literature

In this study, we examined the feasibility of intervention achieved when face-to-face and remote PA follow-up interventions were arranged in series. Although no similar studies have yet investigated intervention effects in this way, research protocols have begun to appear [19,20,21]. There is a high possibility that comparable research findings will emerge in the future. The present study has already produced some results regarding intervention feasibility and is thus advanced.

In this study, the interventions were planned with an emphasis on the two aspects of lifestyle-related disease prevention and care prevention for healthy adults. Therefore, important endpoints were weight loss and indicators of need for care (KCL score in this study). Weight loss was reduced by 5% from initial weight, and KCL scores were targeted to be further reduced to reduce the likelihood of long-term care. Changes due to actual interventions resulted in a 6.3% decrease in body weight after week 15 and a significant decrease in KCL score from 3.0 ± 2.4 at week 1 to 1.8 ± 1.9 at week 15, improving the two major outcomes.

While no other studies to the best of our knowledge have investigated the feasibility of the particular combination of face-to-face and remote interventions examined in this study, research has been conducted on the intervention effects of remote lifestyle-related interventions alone. A recent report showed that computer-tailored interventions via the internet increased accelerometer-assessed MVPA (193 ± 181 min/week to 204 ± 184 min/week) after 6 months in the intervention group, but no significant difference was observed before or after the intervention, or in comparison with the control group without intervention [22]. The average MVPA during remote intervention in the present study was 52.8 ± 38.4 min/day, or 369.6 min/week, which is excellent when compared to the increase in physical activity reported by Volders et al. [22].

Previous studies have regarded the high dropout rate associated with remote interventions as a particular concern, finding dropout rates as high as 30.2% [2] and 9.7% [23] among middle-aged adults during 12-week interventions, and 75.9% among coronary artery disease rehabilitation patients during a 3-month intervention [24]. In this study, all participants completed the 15-week intervention, and there were no dropouts. We believe that the intervention was useful in that respect as well.

### 4.3. Study Strengths

This study has several unique research strengths. First, it answers the question of whether the positive outcomes of face-to-face interventions may be maintained or improved by remote PA follow-up intervention. To the best of our knowledge, no study has attempted to line up face-to-face and remote interventions sequentially, although many have investigated their use in parallel.

Second, this study has the advantage of using a remote behavior change support system that functionally implements ICT and mobile technology. In previous intervention studies, it took a substantial amount of manpower to obtain data on PA and ST. By contrast, the Fitbit data in this study could be automatically obtained from each participant’s smartphone app. In considering the possibilities for business development, the implementation of an abbreviated system that does not require human labor is essential.

### 4.4. Study Limitations

The present study has some research limitations.

First, we should have compared the remote follow-up intervention group with the no intervention group for clearer conclusions, but it was difficult to set up a no intervention group in this joint study with the government office.

Second, this study presents significant issues regarding sample size and generalization. The number of participants was extremely small, and it will be necessary to consider the corresponding statistical analysis. The participants of this study were healthy adults aged 50 to 69 years and could not be generalized to adults with diseases of the same age.

Third, the duration of the intervention was relatively short, and the possible results of longer-term interventions cannot be predicted. Further research is necessary to assess the effectiveness of remote interventions with extended durations.

Fourth, PA and ST before the face-to-face interventions were not evaluated. Thus, it was unclear if the face-to-face interventions improved PA and ST. In this study, we considered a non-inferiority test to examine whether PA deteriorated between the face-to-face and remote follow-up intervention phases, and face-to-face intervention may be clearly superior in promoting PA. In the future, PA should be evaluated before beginning the lifestyle intervention.

Finally, it is also a limitation that it was not possible to evaluate dietary intakes. Not only is dietary assessment non-trivial, but it is also essential to assess if remote interventions are more effective at changing PA or eating behavior.

## 5. Conclusions

In this study, we examined the feasibility of remote PA follow-up intervention after face-to-face intervention in healthy middle-aged adults. The results showed that remote PA follow-up intervention may be feasible and effective in maintaining PA. Furthermore, they suggested that positive effects on weight loss and physical function of face-to-face interventions may be further strengthened remotely. This study is the first attempt to evaluate the feasibility of arranging face-to-face and remote PA interventions in series.

## Figures and Tables

**Table 1:** Baseline characteristics between BCI3 and BCI4 groups.

		Overall (*n* = 30)		BCI3 Group (*n* = 15)		BCI4 Group (*n* = 15)		Student’s *t*-Test	
		Mean	(SD)	Mean	(SD)	Mean	(SD)	*t*-Value	*p*
Body weight (kg)	week 1	64.8	(12.2)	65.0	(11.7)	64.6	(13.0)	0.09	0.927
	week 7	62.0	(11.1)	61.8	(10.9)	62.3	(11.7)	0.11	0.914
	Change	−2.8	(2.5)	−3.2	(3.1)	−2.3	(1.8)	0.93	0.359
Body fat mass (kg)	week 1	19.9	(7.3)	19.4	(5.8)	20.4	(8.7)	0.38	0.707
	week 7	18.4	(6.7)	17.3	(5.6)	19.6	(7.7)	0.92	0.366
	Change	−1.5	(1.8)	−2.1	(2.1)	−0.8	(1.3)	1.95	0.061
Skeletal muscle mass (kg)	week 1	42.4	(9.2)	43.1	(9.3)	41.7	(9.3)	0.41	0.684
	week 7	41.3	(8.6)	42.1	(8.7)	40.4	(8.7)	0.55	0.588
	Change	−1.1	(1.1)	−1.0	(1.2)	−1.3	(1.0)	0.84	0.411
Waist circumference (cm)	week 1	90.6	(10.2)	91.2	(9.4)	90.1	(11.2)	0.30	0.767
	week 7	87.2	(9.5)	87.0	(8.4)	87.5	(10.7)	0.16	0.876
	Change	−3.4	(3.7)	−4.2	(4.7)	−2.6	(2.3)	1.25	0.223
Grip strength (kg)	week 1	32.9	(10.5)	33.8	(9.8)	32.0	(11.4)	0.46	0.649
	week 7	33.6	(10.6)	33.5	(9.7)	33.6	(11.7)	0.03	0.980
	Change	0.7	(3.2)	−0.3	(3.4)	1.6	(2.8)	1.68	0.105
Maximum walking speed	week 1	2.33	(0.37)	2.40	(0.44)	2.26	(0.26)	1.03	0.310
(m/sec)	week 7	2.83	(0.35)	2.95	(0.32)	2.71	(0.35)	2.0	0.056
	Change	0.5	(0.3)	0.6	(0.3)	0.4	(0.3)	0.87	0.390
KCL score (points)	week 1	3.0	(2.4)	3.0	(2.4)	2.9	(2.5)	0.08	0.940
	week 7	2.0	(1.6)	2.5	(1.6)	1.5	(1.4)	1.82	0.080
	Change	−1.0	(2.0)	−0.5	(2.3)	−1.4	(1.7)	1.27	0.214
K6 score (points)	week 1	1.8	(2.2)	1.8	(2.2)	1.7	(2.3)	0.08	0.936
	week 7	1.1	(1.4)	1.1	(1.1)	1.1	(1.7)	0.00	1.000
	Change	−0.7	(1.7)	−0.7	(1.7)	−0.6	(1.8)	0.11	0.916
MVPA (min/day)	weeks 1 to 7	56.3	(38.3)	59.4	(43.3)	53.2	(33.8)	0.43	0.668
LPA (min/day)	weeks 1 to 7	292.4	(80.4)	293.4	(79.2)	291.4	(84.4)	0.07	0.948
ST (min/day)	weeks 1 to 7	700.3	(144.4)	695.8	(112.8)	704.8	(174.4)	0.17	0.868
Walking steps (steps/day)	weeks 1 to 7	12,274	(4063)	12,514	(4612)	12,035	(3578)	0.32	0.753

**Table 2:** Body weight, body compositions, fitness, and psychological factors during face-to-face and remote follow-up interventions.

		Face-to-Face Intervention			Remote Follow-Up Intervention										
		Week 1		Week 7			Week 15			Main Effect (Time)		Main Effect (Group)		Interaction (Time × Group)	
		Mean	(SD)	Mean	(SD)		Mean	(SD)		F	*p*	F	*p*	F	*p*
Body weight (kg)	Overall	64.8	(12.2)	62.0	(11.1)	*	60.7	(11.1)	*#	37.5	<0.001	0.01	0.917	1.57	0.216
	BCI3	65.0	(11.7)	61.8	(10.9)		60.0	(10.9)							
	BCI4	64.6	(13.0)	62.3	(11.7)		61.3	(11.7)							
Body fat mass (kg)	Overall	19.9	(7.3)	18.4	(6.7)	*	17.6	(6.6)	*#	22.1	<0.001	0.66	0.422	3.41	0.040
	BCI3	19.4	(5.8)	17.3	(5.6)		16.2	(5.6)							
	BCI4	20.4	(8.7)	19.6	(7.7)		19.0	(7.4)							
Waist circumference (cm)	Overall	90.6	(10.2)	87.2	(9.5)	*	84.9	(9.2)	*#	48.0	<0.001	0.01	0.918	2.85	0.066
	BCI3	91.2	(9.4)	87.0	(8.4)		84.0	(7.9)							
	BCI4	90.1	(11.2)	87.5	(10.7)		85.7	(10.6)							
Skeletal muscle mass (kg)	Overall	42.4	(9.2)	41.3	(8.6)	*	40.7	(8.5)	*#	36.8	<0.001	0.22	0.640	0.41	0.664
	BCI3	43.1	(9.3)	42.1	(8.7)		41.4	(8.6)							
	BCI4	41.7	(9.3)	40.4	(8.7)		40.0	(8.7)							
Grip strength (kg)	Overall	32.9	(10.5)	33.6	(10.6)		35.0	(10.2)	*	6.72	0.002	0.06	0.807	1.37	0.264
	BCI3	33.8	(9.8)	33.5	(9.7)		35.6	(9.9)							
	BCI4	32.0	(11.4)	33.6	(11.7)		34.4	(10.8)							
Maximum walking speed	Overall	2.33	(0.37)	2.83	(0.35)	*	2.90	(0.49)	*	50.2	0.000	2.94	0.098	0.62	0.540
(m/sec)	BCI3	2.40	(0.44)	2.95	(0.32)		3.03	(0.56)							
	BCI4	2.26	(0.26)	2.71	(0.35)		2.76	(0.39)							
KCL score (points)	Overall	3.0	(2.4)	2.0	(1.6)		1.8	(1.9)	*	5.94	0.005	1.50	0.231	1.22	0.303
	BCI3	3.0	(2.4)	2.5	(1.6)		2.3	(2.3)							
	BCI4	2.9	(2.5)	1.5	(1.4)		1.3	(1.3)							
K6 score (points)	Overall	1.8	(2.2)	1.1	(1.4)		0.6	(1.2)	*	7.19	0.002	0.07	0.792	0.46	0.631
	BCI3	1.8	(2.2)	1.1	(1.1)		0.4	(0.6)							
	BCI4	1.7	(2.3)	1.1	(1.7)		0.9	(1.5)							

* vs. week 1 *p* < 0.05, # vs. week 7 *p* < 0.05.

**Table 3:** Physical activity, sitting time, and walking steps during face-to-face and remote follow-up interventions.

		Face-to-Face Intervention (Weeks 1 to 7)		Remote Follow-Up Intervention (Weeks 8 to 15)		Main Effect (Time)		Main Effect (Group)		Interaction (Time × Group)	
		Mean	(SD)	Mean	(SD)	F	*p*	F	*p*	F	*p*
MVPA (min/day)	Overall	56.3	(38.3)	52.8	(38.4)	1.27	0.270	0.32	0.575	0.30	0.590
	BCI3	59.4	(43.3)	57.6	(41.2)						
	BCI4	53.2	(33.8)	48.1	(36.1)						
LPA (min/day)	Overall	292.4	(80.4)	290.1	(94.3)	0.08	0.775	0.19	0.668	3.84	0.060
	BCI3	293.4	(79.2)	275.6	(98.3)						
	BCI4	291.4	(84.4)	304.7	(91.2)						
ST (min/day)	Overall	700.3	(144.4)	705.2	(151.8)	0.17	0.680	0.06	0.802	3.65	0.066
	BCI3	695.8	(112.8)	723.3	(146.5)						
	BCI4	704.8	(174.4)	687.1	(159.8)						
Walking steps (steps/day)	Overall	12,274	(4063)	11,971	(4306)	0.85	0.364	0.18	0.679	0.22	0.640
	BCI3	12,514	(4612)	12,366	(4853)						
	BCI4	12,035	(3578)	11,576	(3810)						

## Data Availability

The data presented in this study are available on request from the corresponding author.
